# The Pathogenesis of Giant Condyloma Acuminatum (Buschke-Lowenstein Tumor): An Overview

**DOI:** 10.3390/ijms23094547

**Published:** 2022-04-20

**Authors:** Dorota Purzycka-Bohdan, Roman J. Nowicki, Florian Herms, Jean-Laurent Casanova, Sébastien Fouéré, Vivien Béziat

**Affiliations:** 1 Department of Dermatology, Venereology and Allergology, Medical University of Gdansk, 80-214 Gdansk, Poland; rnowicki@gumed.edu.pl; 2 Department of Dermatology, APHP, Saint-Louis Hospital, Université de Paris, 1 Avenue Claude Vellefaux, 75010 Paris, France; florian.herms@aphp.fr (F.H.); sebastien.fouere@aphp.fr (S.F.); 3 Centre for Genital and Sexually Transmitted Diseases, APHP, Saint-Louis Hospital, 75010 Paris, France; 4 Laboratory of Human Genetics of Infectious Diseases, Necker Branch, Institut National de la Santé et de la Recherche Médicale (INSERM) UMR-1163, Necker Hospital for Sick Children, 75015 Paris, France; casanova@rockefeller.edu; 5 St. Giles Laboratory of Human Genetics of Infectious Diseases, Rockefeller Branch, Rockefeller University, New York, NY 10065, USA; 6 Imagine Institute, University of Paris Cité, 75015 Paris, France; 7 Department of Pediatrics, Necker Hospital for Sick Children, AP-HP, 75015 Paris, France; 8 Howard Hughes Medical Institute, New York, NY 10065, USA

**Keywords:** giant condyloma acuminatum, Buschke-Lowenstein tumor, human papillomavirus, immunodeficiency, genetics

## Abstract

Giant condyloma acuminatum, also known as Buschke-Lowenstein tumor (BLT), is a rare disease of the anogenital region. BLT is considered a locally aggressive tumor of benign histological appearance, but with the potential for destructive growth and high recurrence rates. BLT development is strongly associated with infection with low-risk human papillomaviruses (HPVs), mostly HPV-6 and -11. Immunity to HPVs plays a crucial role in the natural control of various HPV-induced lesions. Large condyloma acuminata are frequently reported in patients with primary (e.g., DOCK8 or SPINK5 deficiencies) and secondary (e.g., AIDS, solid organ transplantation) immune defects. Individuals with extensive anogenital warts, including BLT in particular, should therefore be tested for inherited or acquired immunodeficiency. Research into the genetic basis of unexplained cases is warranted. An understanding of the etiology of BLT would lead to improvements in its management. This review focuses on the role of underlying HPV infections, and human genetic and immunological determinants of BLT.

## 1. Introduction

Papillomaviruses (PVs) are small circular double-stranded DNA viruses from the family Papillomaviridae. PVs are highly host-specific, and display preferential tropism for squamous stratified epithelia, including skin, and conjunctival, oral, and anogenital mucosae [1]. Human PVs (HPVs) are classified into five main genera (α-, β-, γ-, µ-, and ν-HPVs) on the basis of their DNA sequences. To date, almost 450 HPV genotypes have been isolated and sequenced [2]. According to serological data, most humans have been exposed to HPVs, and HPV infection is one of the most common sexually transmitted diseases [3,4]. Epidemiological studies have shown the risk of anogenital HPV infections to be positively associated with number of sexual partners and inversely associated with age at first sexual intercourse [5,6]. Infection is usually asymptomatic or self-limiting. However, in some individuals, insufficient immune control of viral infection leads to persistent lesions, profuse warts, dysplasia, or cancer development [6,7].

The α-HPVs include species with cutaneous (e.g., HPV-2, -27, and -57) and mucous membranes (e.g., HPV-6, -11, -16, and -18) tropism [1]. Based on their oncogenic potential they are further divided in low- and high-risk HPVs. Infection with low-risk HPV types usually remains asymptomatic or benign, and lesions regress spontaneously after a few weeks or months. Low-risk α-HPVs can cause common warts but may also be responsible for tree man syndrome (TMS), as reported in several patients [8,9]. TMS is characterized by persistent giant cutaneous horns. Other low-risk α-HPVs, such as HPV-6 and HPV-11, are associated with condylomas, but may contribute to the formation of giant condyloma acuminata with destructive local progression [10]. High-risk α-HPVs are HPV-16, -18, -31, -33, -35, -39, -45, -51, -52, -56, -58, -59, -66, -68, and -70. Persistent infection with high-risk HPV types is the main risk factor of developing HPV-induced malignancies. In particular, HPV-16 is responsible for the majority of HPV-induced cervical cancer, oropharyngeal and anogenital squamous cell carcinoma (SCC) [11,12,13]. HPV types belonging to genus beta have a cutaneous tropism and typically cause asymptomatic infections in the general population. Patients with epidermodysplasia verruciformis (EV), a rare condition, present with profuse flat warts or scaly, reddish, brownish, or achromic plaques due to an isolated susceptibility to β-HPVs infection, and sometimes HPV-3 from the α genus [14]. In these patients, β-HPVs with a high oncogenic potential, such as HPV-5, are co-factors of cutaneous SCC development [14,15,16]. Finally, the γ-, µ-, and ν-HPVs all present skin tropism and are associated with benign common warts (e.g., HPV-4, a γ-HPV) or plantar myrmecia (e.g., HPV-1, a µ-HPV) [17].

Extensive HPV lesions of all types are frequently observed in individuals on immunosuppressive therapy or with human immunodeficiency virus (HIV) infection, suggesting a crucial role of CD4^+^ T cells in controlling HPVs [18]. Severe isolated or syndromic (associated with other infections) HPV lesions are also observed in individuals with inborn errors of immunity [18,19]. For instance, the isolated susceptibility of EV patients to β-HPVs results from inherited EVER1 (encoded by *TMC6*), EVER2 (encoded by *TMC8*), or calcium- and integrin-binding protein 1 (CIB1) deficiency [20,21]. Syndromic EV results from mutations in genes involved in T cell immunity (e.g., *STK4*, *RHOH*) [17]. Mutations of certain immune response-related genes, such as *GATA2, CXCR4,* and *DOCK8,* are associated with a greater predisposition to multiple types of HPV-induced lesions, including extensive anogenital warts [19]. Moreover, recent studies have shown that the defective CD28 signaling pathway can also decrease the anti-α- and γ-HPV responses, thereby increasing the risk of extensive warts and condylomas [8,22]. This review explores the roles of HPV infection, immunodeficiencies, and host genetics in the pathogenesis of anogenital HPV lesions, with particular focus on giant condyloma acuminatum.

## 2. Giant Condyloma Acuminatum (Buschke-Lowenstein Tumor)—A Short Description of the Disease

### 2.1. Classification

Giant condyloma acuminatum is also known as Buschke-Lowenstein tumor (BLT). BLT was first reported in 1896, in Neisser’s Sterokopischer Atlas, by Abraham Buschke [23]. Subsequently, in 1925, Abraham Buschke and his assistant Ludwig Löwenstein described BLT as a penile lesion clinically resembling both common condylomas and SCC, but with a different histological appearance and biological behavior [24,25]. BLT is considered to be a locally aggressive tumor characterized by (i) benign histology, (ii) a potential for destructive growth, (iii) malignant transformation (estimated rate of 56%) without propensity for metastasis, and (iv) with a high rate of recurrence after excision (66%) and an overall mortality of approximately 20% [26]. Some authors consider BLT to be intermediate between condyloma acuminatum and SCC, whereas others classify it as an anogenital verrucous carcinoma (a well-differentiated type of SCC) [27,28]. According to recent studies, BLT and verrucous carcinoma should be recognized as two separate entities [29,30]. Indeed, BLTs are associated with low-risk HPV-6 or HPV-11 (see Section 3), whereas verrucous carcinomas are not usually HPV-driven [30,31].

### 2.2. Clinical Presentation

BLT tends to occur in individuals in their forties. It has an estimated incidence of about 0.1% in the general population and a male-to-female ratio of 2.7:1 [28,32,33]. However, its precise incidence is unknown. It presents as a slow-growing cauliflower-like mass in the genital or anorectal area, with relatively slow infiltration into deeper tissues [26]. The disease starts from a long-standing condyloma acuminatum, which can grow to sizes of more than 10 cm in diameter. Progression from the first symptoms of condyloma to BLT development may take 2.8–9.6 years, or longer [26]. Tumor growth is usually slow, but may be rapid in immunocompromised individuals [34,35]. Non-sexual transmission via fomites is possible, but cases of condyloma acuminata and BLT in children should always raise the suspicion of sexual abuse, for which both medical and social evaluation is essential [36]. Unusual clinical presentations of BLT with rapid growth may suggest malignant transformation [28].

### 2.3. Diagnosis

**Figure 1:**
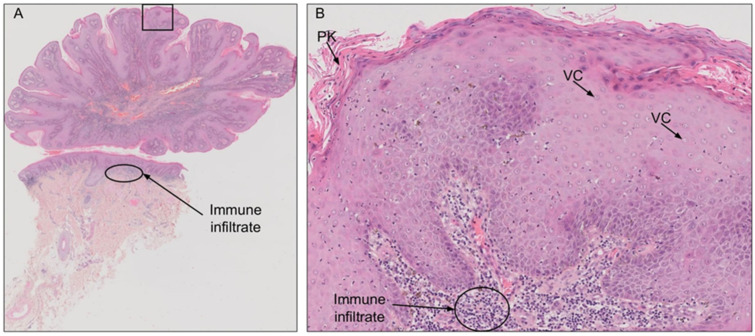
Hematoxylin and eosin staining of a penile Buschke-Lowenstein tumor (BLT). (**A**) BLT presents with a typical cauliflower shape, without invasion of the dermis. (**B**) Magnification of the indicated area from A. The histological features are typical of HPV infection, with numerous vacuolated cells (VC; koilocytosis) and parakeratosis (PK). Large immune infiltrates are visible in the dermis downstream from the lesion.

BLT is diagnosed on the basis of patient history, clinical and histological presentation. Histologically, the tumor is characterized by papillomatosis, hyperkeratosis, parakeratosis, acanthosis, and koilocytosis (Figure 1) [34]. Careful histological examination is crucial to exclude transformation to SCC. Imaging studies, such as computed tomography and magnetic resonance imaging, are strongly recommended for assessment of the local and regional extension and to ensure that optimal therapy is prescribed [37].

### 2.4. Treatment

A wide radical excision, followed by reconstructive surgery, seems to be the optimal therapeutic strategy for BLT management [38]. However, for extensive tumors, preoperative chemotherapy or radiotherapy can be used to promote tumor shrinkage, rendering the debulking procedure safer [39]. Follow-up visits are necessary due to the high risk of recurrence (estimated at more than 60%) [26]. BLT-related mortality appears to be mostly associated with infectious complications [29]. The maintenance of good hygiene and the correct healing of postoperative wounds are, therefore, essential to reduce the risk of death. Immunomodulatory treatments have also been tested in clinical trials. Topical imiquimod, which induces interferon alpha (IFN)*-*α production upon binding to Toll-like receptor 7 (TLR7), has shown benefit in the treatment of BLT [40,41,42,43]. Interestingly, Geusau et al. reported a regression of deeply infiltrating BLT following long-term intra-lesional IFN-α2b therapy [44]. Unfortunately, less favorable outcomes were reported in other studies after imiquimod or IFN-α treatment [45,46,47]. IFN-α exerts antiviral activity by inducing the expression of protective genes with products that inhibit viral replication and reduce viral dissemination. However, patients with inherited IFNAR1, IFNAR2, IFNGR1, or IFNGR2 deficiencies are not susceptible to severe HPV infections, suggesting that type I and II interferons are not central to HPV-disease pathogenesis [48,49,50]. Similarly, epidermal growth factor receptor (EGFR) overexpression in benign condyloma acuminata may play a role in dysplastic cell proliferation. This led Bowman et al. to implement systemic chemotherapy including the EGFR inhibitor cetuximab in a patient with metastatic BLT, resulting in a partial response for eight months [51]. Finally, HPV quadrivalent and nonavalent vaccines (Gardasil vaccines) significantly decrease the incidence of genital warts [52]. Although no studies measured the impact of vaccination on BLT incidence, HPV vaccines are also likely to decrease the risk of BLT given that the two causal HPV types, HPV-6 and HPV-11, are included in these vaccines. In addition, some authors suggested their possible therapeutic properties and presented clinical cases of regression of giant condyloma acuminata after HPV vaccination [53,54]. These promising observations show that more studies are required to evaluate the therapeutic value of HPV vaccines and other immunomodulatory treatments against BLT. In summary, surgery is the only really effective treatment of BLT to date, but less invasive procedures may also be beneficial.

## 3. The Role of Low-Risk HPVs in BLT Pathogenesis

Approximately 90% of genital warts are caused by HPV-6 or -11 [55], with HPV-6 predominating [56]. Low-risk HPVs have a low transformation capacity. Despite their high prevalence in genital warts, DNA from HPV-6 and HPV-11 were found only in 4% and 3% of anal cancers, respectively [57], and <1% of cervical cancers [58]. In contrast, HPV-6 and HPV-11 are found in most, if not all, BLT cases, demonstrating a key role for these viruses in tumor development [37,38,59,60,61]. An excellent review was previously dedicated to the pathogenicity and carcinogenicity of low-risk HPVs compared to high-risk HPVs [62]. It is believed that irrespective of high- or low-risk status, in the course of persistent infection, HPVs make common changes to the infected cells, and that there is convergence in the pathways that they affect [62]. Among a large number of biological activities, high-risk HPV E6 and E7 oncoproteins induce degradation of the tumor suppressor protein p53 and inhibit the retinoblastoma protein (pRb), respectively [62,63,64,65]. This leads to abnormal proliferation from the earliest layer of the epithelium, and oncogenic transformation of infected host cells. In contrast, low-risk α-HPV do not induce cell proliferation in the basal and parabasal layer of the epithelium, contributing to their lower oncogenic potential [62]. This is probably explained by difference in biological activity of E6 and E7 proteins from low- and high-risk HPVs. The overexpression of E6 or E7 from low-risk HPV-6 or HPV-11 in various cellular models impact the expression of numerous host genes [66,67]. Unlike E6 from high-risk HPV-16, E6 from low-risk HPV-11 induces p53 degradation in a cell density-dependent manner [68]. E7 from low-risk HPVs targets pRb family members similarly to high-risk HPVs; however, they were shown to have a preference for p130 which regulates cell cycle entry in the upper epithelial layers [62,69].

While malignant cell transformation in response to high-risk HPV infection has been extensively studied and well characterized, little is known about the mechanisms involved in the progression of low-risk HPV-driven benign condyloma acuminatum to the BLT phenotype. To our knowledge, no studies investigated the viral protein expression patterns, in particular E6 and E7, in BLT compared to conventional condyloma. Nevertheless, the role of p53 in the malignant progression of BLT was highlighted by Pilotti et al., based on immunocytochemical and molecular data [70]. These authors studied five cases of vulvar verrucous carcinoma and two cases of BLT associated with invasive SCC. Neither p53 overexpression nor HPVs were detected in verrucous carcinoma samples, whereas both cases of BLT with invasive SCC tested positive for HPV-6 or -11 and presented p53 overexpression in nuclei. Interestingly, the malignant area of one of these BLT with SCC carried a pathogenic *TP53* missense mutation (Gly245Ser) in the DNA-binding domain of the p53 protein [70]. Altogether, while it has been known for a long time that HPV-6 or HPV-11 are required for BLT development, more studies are needed to understand the mechanisms underlying the transition between benign condyloma and BLT.

## 4. Possible Impact of Viral Genome Rearrangements, Mutations, and Host Genome Integration on BLT Development

In normal conditions, HPVs exist as episomes in infected cells. During tumorigenesis, high-risk HPVs can integrate into the host genome, but they may sometimes remain episomal [71,72]. Integration disrupts the viral E2 gene and thus leads to dysregulation of viral E6 and E7 oncogene expression that promotes cell proliferation, abolishes cell-cycle checkpoints, and causes progressive genetic instability [71]. By contrast, probably reflecting their low oncogenic potential [73], low-risk HPVs, such as HPV-6 and HPV-11, do not usually integrate into host DNA, including in BLT [74,75], anal [76], cervical [58], and vulvar cancers [77]. To our knowledge, HPV-6 was never reported integrated in BLT, and only once in an anogenital cancer [78]. However, HPV-6 and HPV-11 integration was reported in some patients with head and neck cancers [79,80,81]. Altogether, the available data strongly suggest that HPV-6 or HPV-11 integration is not required for BLT development. This is reminiscent of episomal HPV-2 in a TMS case [8], another devastating benign cutaneous tumor driven by a low-risk HPV.

Instead of viral integration, mutations or rearrangement within the episomal virus may explain BLT development. Rearrangements within the upstream regulatory region (URR) of HPV-6 or HPV-11 from BLT were reported in multiple studies [75,82,83,84,85]. Duplications within the URR have also been detected in anogenital carcinomas associated with HPV-16 [86], laryngeal carcinomas containing HPV-11 [87], and one SCC of the lung [88] and two SCCs of the vulva related to HPV-6 infection [89,90]. It has been suggested that URR duplications may increase the otherwise low oncogenic potential of HPV-6 and HPV-11 by enhancing transcription of the transforming genes E6 and E7 [91,92]. However, Rübben et al. showed that host factors were probably more responsible for BLTs, with rearrangements of the URR of HPVs probably representing only secondary events in BLT development, as such rearrangements are also detected in benign genital warts [82]. In addition, to our knowledge, no nucleotide substitution identified in the HPV-6 or HPV-11 strains from BLT samples can explain tumorigenesis [75,82,83,93]. However, the full-length DNA sequence of the HPV strain within the BLT was not obtained for the overwhelming majority of reported cases. Further studies are required to determine whether mutations or viral genome rearrangements can influence the development of BLT and possible neoplastic transformation. This would require a systematic report of the full-length HPV sequence, as well as of the viral genome integration status in BLT.

## 5. Secondary Immunodeficiencies in the Etiology of BLT

Cell-mediated immune response is crucial for control of HPV-induced lesions [94]. Indeed, patients on immunosuppressive drugs, and HIV-infected patients have a higher risk of severe HPV infections than the general population [95,96,97]. CD4^+^ T cells as well as monocytes/macrophages prevail within regressing condylomas [44,98,99]. Akinboro et al. reported lower blood CD4^+^ cell counts in HIV-positive patients with genital warts than those without such lesions (101 cells/µL vs. 294 cells/µL, respectively) [100]. They also found that the extent of the viral warts was correlated with CD4^+^ T-cell count [100]. In this context, not surprisingly, BLT is more frequent in individuals with HIV infection [101,102,103,104,105] or on immunosuppressive drugs, such as patients with a history of stem cell or solid organ transplantation [106,107,108,109]. Risk of rapid progression of BLT into metastatic SCCs in HIV-infected patients is elevated [61,110]. Grodner et al. reported an improvement in voluminous pelvic BLT after highly active antiretroviral therapy alone in an HIV-infected patient [111]. Surgical excision of the BLT was initially planned but was postponed when significant regression of the tumor was observed on antiretroviral therapy, together with CD4 immune recovery (gradual increase in CD4^+^ T-cell count from 26 cells/µL to 229 cells/mm^3^ over a period of six months) and the suppression of HIV-1 replication (HIV RNA levels decreased from 5.21 log copies/mL to <20 copies/mL and remained undetectable thereafter). Given the absence of any other therapy in this patient, the authors concluded that cellular immune recovery after antiretroviral therapy alone was responsible for the regression of BLT. Thus, acquired immunodeficiencies should be considered in all cases of extensive HPV lesions, including BLT, to improve patient outcomes.

## 6. Leading Genetic Causes of Susceptibility to Extensive Anogenital HPV Lesions

Mutations in several immunity-related genes have been associated with extensive condyloma acuminata (Table 1).

The genetic predispositions to common warts and anogenital HPV lesions overlap considerably [17]. Gain-of-function (GOF) mutations of the *CXCR4* gene encoding the receptor of the CXCL12 chemokine are responsible for WHIM syndrome (HPV-induced warts, hypogammaglobulinemia, recurrent bacterial infections, and myelokathexis) [114]. Patients with WHIM syndrome are particularly susceptible to extensive warts on the hands, feet, and trunk. They may also develop genital and anal condyloma acuminata, and female patients may develop vulval and cervical dysplasia [115,132]. Monoallelic missense and null mutations of *GATA2* lead to a deficiency in an important transcription factor for hematopoiesis and maintenance of the stem-cell compartment (GATA2) [19]. In addition to its role in myelodysplasia and leukemia, GATA2 deficiency increases the risk of profuse and recurrent cutaneous or anogenital warts [124,125,133]. Difficulties in treatment of generalized unremitting warts are also observed in individuals with autosomal recessive dedicator of cytokinesis 8 (DOCK8) deficiency [119,120]. DOCK8 is essential for the maintenance of T-cell integrity in collagen-dense tissues, and this translates into poor defense against pathogens in the absence of DOCK8 [134]. DOCK8 is also important for dendritic cell migration to lymph nodes [135]. Venegas-Montoya et al. reported a six-year-old DOCK8-deficient patient with disseminated flat warts, who also presented an extensive condyloma acuminate around the scrotum and groin folds [121]. Overall, mutations of *CXCR4*, *GATA2*, and *DOCK8* result in low numbers of both antigen-presenting cells and T cells. Thus, inborn errors of immunity simultaneously impairing these two arms of immunity underlie extreme penetrance of cutaneous and anogenital HPV infections.

## 7. Significance of the CD28 Axis in the Development of HPV-Related Anogenital Lesions

Individuals with autosomal recessive CARMIL2 deficiency present a wide spectrum of clinical phenotypes, with bacterial, fungal, and viral infections, including anogenital condylomas in some patients [22,113]. CARMIL2 is a protein involved in the CD28 cosignaling of T cells, and in cytoskeletal organization and cell migration [112]. The discovery of severe cutaneous HPV infections in patients with CD28 deficiency suggested that defective CD28 signaling in T cells was the main driver of HPV susceptibility in individuals with *CARMIL2* mutations [8]. However, in accordance with the incomplete penetrance of HPV infection in CARMIL2-deficient patients, the three reported CD28-deficient patients developed no anogenital lesions, despite one patient being seropositive for HPV-6 and HPV-11 [8]. Consistent with the hypothesis that the CD28 axis plays a crucial role in the anti-HPV response, mutations of the caspase activation and recruitment domain 11 (*CARD11*) and magnesium transporter 1 (*MAGT1*) genes also increase the risk of severe HPV infections [128,136]. CARD11 is a scaffolding protein required for antigen receptor-induced NF-κB activation, notably downstream from CD28. MAGT1 deficiency is a congenital disorder of glycosylation. MAGT1 is crucial for the glycosylation and cell-surface expression of major immune receptors, including CD28. It has been reported that 27% of patients carrying dominant-negative mutations of *CARD11* suffer from unspecified skin warts [136], whereas, in individuals with MAGT1 deficiency, flat warts, predominantly affecting the palms and soles, are observed in 30% of cases, and some patients develop extensive perineal condyloma acuminata [128]. Thus, the CD28 pathway probably contributes to anogenital HPV control, and the early pathogenesis of BLT.

## 8. Other Genes in the Pathogenesis of Anogenital HPV-Induced Lesions

There are few reports concerning other genes involved in predisposition to anogenital HPV infection. Individuals with Netherton syndrome, an autosomal recessive ichthyosis caused by mutations of *SPINK5*, are prone to the development of giant warts [129,130]. Ashton et al. published a pediatric case of BLT in the natal cleft in a patient with Netherton syndrome [130]. Extensive vulvovaginal and perianal warts, including BLT, have been reported in patients with integrin B2 (*ITGB2*) or DNA cross-link repair 1C gene (*DCLRE1C)* deficiencies [17,19]. Similarly, patients with autosomal recessive deficiency of the zeta chain-associated protein of 70 kDa (ZAP70) may present severe HPV infections. Chinn et al. published a case report concerning a ZAP70-deficient woman with recurrent oral and cutaneous warts and HPV-induced cervical dysplasia [137]. Severe anogenital manifestations of HPV infection have also been observed in individuals with autosomal recessive inducible costimulator (ICOS) pathway deficiency. Schepp et al. reported HPV-induced vulvar carcinoma in one ICOS-deficient patient [138], whereas Roussel et al. recently reported the case of a male patient with an autosomal recessive mutation of the inducible T-cell costimulator ligand gene (*ICOSLG*) [126]. This last patient was 16 years old, and subsequently developed recurrent genital warts that spread, over the years, to involve the scrotum, perineum, perianal, and inguinal regions [126]. To our knowledge, there are no published case reports of familial BLT (multiple cases in the same family). Nevertheless, there is strong evidence that inborn errors of immunity can underlie severe anogenital HPV infections, including BLT.

The Table 2 summarizes all discussed genes and the function of encoded proteins.

## 9. Conclusions

The prognosis of BLT probably depends on tumor size, SCC transformation, local recurrence, secondary infections, and associated immunodeficiencies. Early diagnosis and appropriate aggressive therapy may reduce both medical and surgical morbidity, and overall mortality. Primary or secondary immunodeficiencies should be suspected in individuals with BLT. In the absence of acquired immunodeficiency, genetic investigations should be envisaged. Recent studies have shown that inborn errors of immunity conferring a predisposition to common warts and anogenital HPV lesions largely overlap, and that they impair host cellular immunity, including that mediated by CD4^+^ T cells, in particular. The smaller number of reports of severe anogenital warts than of cutaneous warts in patients with primary immunodeficiencies probably reflects the epidemiology of the disease, with a peak incidence in childhood for cutaneous warts, and during the third decade of life for anogenital warts [139,140]. Inborn errors of immunity conferring predisposition to various infections, including those caused by HPVs, probably manifest before exposure to sexually transmissible HPVs. As a result, prophylactic measures or a severe course of immunodeficiency (e.g., early death, transplantation) probably reduce the incidence of anogenital HPV lesions in such patients. Despite the large body of knowledge available regarding HPV infections, further immunological and genetic investigations into susceptibility to severe and persistent HPV lesions of the anogenital region are required. The study of patients with isolated severe anogenital HPV infections is of particular interest, and may unravel important molecular pathways, as recently exemplified by the discovery of CD28 deficiency in patients with tree man syndrome [8]. Such efforts should lead to improvements in the clinical management of patients.

## Figures and Tables

**Table 1:** Primary immunodeficiencies associated with extensive condyloma acuminata.

Primary Immunodeficiency	Gene Mutated	Inheritance	Phenotype	References
CARMIL2 deficiency	*CARMIL2* *(RLTPR)*	AR	Common warts, recurrent condylomas, broad susceptibility to infection, immune dysregulation, EBV-driven smooth muscle tumors	[17,22,112,113]
WHIM syndrome	*CXCR4*	AD	Common warts, condyloma acuminata, hypogammaglobulinemia (low IgG and IgA, normal IgM), infections, myelokathexis	[114,115,116]
DCLRE1C deficiency	*DCLRE1C*	AR (hypomorphic)	Extensive HPV-related anogenital lesions, atypical EV, low numbers of B cells, hypogammaglobulinemia	[117,118]
DOCK8 deficiency	*DOCK8*	AR	Common warts, condyloma acuminata, atypical EV, other viral cutaneous infections (VZV, HSV, molluscum contagiosum), eczema, food allergy, asthma, allergic rhinitis, bacterial pneumonia, candidiasis, abscesses, cancer, thrombocytosis, eosinophilia, lymphopenia	[119,120,121]
GATA2 deficiency [DCML, MDS, MonoMAC syndrome, WILD syndrome, Emberger syndrome]	*GATA2*	AD	Common warts, condyloma acuminata, VZV, HSV, fungal infections, lymphedema, myelodysplasia, leukemia, panniculitis, cancer, low B-cell levels	[19,122,123,124,125]
ICOSL deficiency	*ICOSLG*	AR	Common warts, extensive condyloma acuminata, orolabial HSV infections, angular cheilitis, mouth ulcers, hypogammaglobulinemia, neutropenia, lymphopenia	[126]
LAD-1	*ITGB2*	AR	Extensive common warts and condyloma acuminata, frequent systemic, skin, and soft tissue infections, inflammatory bowel disease, impaired wound healing, gingivitis, periodontitis	[19,127]
MAGT-1 deficiency XMEN syndrome	*MAGT-1*	XLR	Cutaneous warts, perineal condylomas, EBV infections, infections of the ear and nose, viral infections of the skin, cancers	[17,128]
Netherton syndrome	*SPINK5*	AR	Common warts, giant condyloma acuminata, ichthyosis, eczema, bamboo hair, asthma, food allergy, high IgE levels	[129,130]
WAS	*WAS*	XLR	Common warts, condyloma acuminata, thrombocytopenia, infections, eczema, cancers, autoimmune manifestations	[17,131]

CARMIL2, capping protein regulator and myosin 1 linker 2; AR, autosomal recessive; WHIM, warts, hypogammaglobulinemia, infections, and myelokathexis; *CXCR4*, CXC chemokine receptor 4; AD, autosomal dominant; DCLRE1C, DNA cross-link repair 1C; DOCK8, dedicator of cytokinesis 8; VZV, varicella zoster virus; HSV, herpes simplex virus; GATA2, GATA-binding protein 2; DCML, dendritic cell, monocyte, B and NK lymphoid deficiency; MDS, deafness, lymphedema, mononuclear cytopenia, infection, myelodysplasia; MonoMAC, monocytopenia and mycobacterial infection syndrome; WILD, warts, immunodeficiency, lymphedema, dysplasia; *ICOSLG*, inducible T-cell costimulator ligand; LAD-1, leukocyte adhesion deficiency type-1; *ITGB2*, integrin B2; MAGT-1, magnesium transporter 1; XMEN, X-linked immunodeficiency with magnesium defect, Epstein-Barr virus infection, and neoplasia syndrome; XLR, X-linked recessive; EBV, Epstein-Barr virus; *SPINK5*, serine protease inhibitor Kazal-type 5; WAS, Wiskott Aldrich syndrome.

**Table 2:** Summary of the discussed genes.

Gene (Official Symbol)	Gene (Official Full Name)	Function of Encoded Protein
*CARD11*	caspase activation and recruitment domain 11	a member of the membrane-associated guanylate kinase (MAGUK) family; plays a key role in adaptive immune response by transducing the activation of NF-kappa-B downstream of T-cell receptor and B-cell receptor engagement
*CARMIL2*	capping protein regulator and myosin 1 linker 2	a member of CARMIL family of proteins; involved in the CD28 cosignaling of T cells, and in cytoskeletal organization and cell migration
*CIB1*	calcium and integrin binding 1	regulator of diverse cellular processes including migration, adhesion, proliferation, and cell death/survival. CIB1 deficiency is associated with epidermodysplasia verruciformis
*CXCR4*	C-X-C chemokine receptor type 4	a receptor of the CXCL12 chemokine; is involved in multiple signaling pathways that orchestrate cell migration, hematopoiesis and cell homing, and retention in the bone marrow
*DCLRE1C*	DNA cross-link repair 1C	a nuclear protein; regulation of the cell cycle in response to DNA damage, and TCR and BCR recombination
*DOCK8*	dedicator of cytokinesis 8	a member of the DOCK180 family of guanine nucleotide exchange factors; critical role in cell migration and survival of several types of immune system cells
*GATA2*	GATA-binding protein 2	a member of the GATA family of zinc-finger transcription factors; plays a critical role in maintaining the pool of early hematopoietic cells
*ICOS*	inducible T cell costimulator	protein belonging to the CD28 and CTLA-4 cell-surface receptor family; T cell co-activating receptor, involved in T cell immune responses
*ICOSLG*	inducible T cell costimulator ligand	ligand of ICOS, involved in T cell immune responses
*IFNAR1*	interferon alpha and beta receptor subunit 1	forms one of the two chains of a receptor for IFN-α and IFN-β; involved in immune response; functions as an antiviral factor
*IFNAR2*	interferon alpha and beta receptor subunit 2	forms one of the two chains of a receptor for IFN-α and IFN-β; involved in immune response; functions as an antiviral factor
*IFNGR1*	interferon gamma receptor 1	the ligand-binding chain (alpha) of the gamma interferon receptor; non-redundant roles against intra-cellular pathogens (in particular mycobacteria)
*IFNGR2*	interferon gamma receptor 2	the non-ligand-binding beta chain of the gamma interferon receptor. non-redundant roles against intra-cellular pathogens (in particular mycobacteria)
*ITGB2*	integrin subunit beta 2	an integrin beta chain; participate in cell adhesion as well as cell-surface mediated signaling
*MAGT1*	magnesium transporter 1	a ubiquitously expressed magnesium cation transporter protein; crucial for the glycosylation and cell-surface expression of major immune receptors, including CD28
*SPINK5*	serine peptidase inhibitor Kazal type 5	lympho-epithelial Kazal-type related inhibitor (LEKT1); plays a role in skin and hair morphogenesis, as well as anti-inflammatory and antimicrobial protection of mucous epithelia
*TMC6* *(EVER1)*	transmembrane channel like 6	integral membrane protein located in the endoplasmic reticulum; predicted to form transmembrane channels; TMC6 deficiency is associated with epidermodysplasia verruciformis
*TMC8* *(EVER2)*	transmembrane channel like 8	integral membrane protein located in the endoplasmic reticulum; predicted to form transmembrane channels; TMC8 deficiency is associated with epidermodysplasia verruciformis
*ZAP70*	zeta chain of T cell receptor associated protein kinase 70	an enzyme belonging to the protein tyrosine kinase family; plays a role in T-cell development and lymphocyte activation; essential for thymocyte development

## Data Availability

Not applicable.

## References

[1] Altamura G., Tommasino M., Borzacchiello G. (2020). Cutaneous vs. Mucosal Tropism: The Papillomavirus Paradigm Comes to an “and”. Front. Microbiol..

[2] McBride A.A. (2021). Human papillomaviruses: Diversity, infection and host interactions. Nat. Rev. Microbiol..

[3] Rahman S., Campbell C.M.P., Waterboer T., Rollison D.E., Ingles D.J., Torres B.N., Michel A., Sudenga S.L., Pawlita M., Villa L.L. (2016). Seroprevalence of cutaneous human papillomaviruses (HPVs) among men in the multinational HPV Infection in Men study. J. Gen. Virol..

[4] Antonsson A., Green A.C., Mallitt K.-A., O’Rourke P.K., Pandeya N., Pawlita M., Waterboer T., Neale R.E. (2010). Prevalence and stability of antibodies to 37 human papillomavirus types—A population-based longitudinal study. Virology.

[5] Tulay P., Serakinci N. (2016). The role of human papillomaviruses in cancer progression. J. Cancer Metastasis Treat..

[6] Manini I., Montomoli E. (2018). Epidemiology and prevention of Human Papillomavirus. Ann Ig.

[7] de Sanjosé S., Bruni L., Alemany L. (2014). HPV in genital cancers (at the exception of cervical cancer) and anal cancers. Presse Médicale.

[8] Béziat V., Rapaport F., Hu J., Titeux M., des Claustres M.B., Bourgey M., Griffin H., Bandet É., Ma C.S., Sherkat R. (2021). Humans with inherited T cell CD28 deficiency are susceptible to skin papillomaviruses but are otherwise healthy. Cell.

[9] Wang W., Wang C., Xu S., Chen C., Tong X., Liang Y., Dong X., Lei Y., Zheng X., Yu J. (2007). Detection of HPV-2 and identification of novel mutations by whole genome sequencing from biopsies of two patients with multiple cutaneous horns. J. Clin. Virol..

[10] Pennycook K.B., McCready T.A. (2021). Condyloma Acuminata. StatPearls.

[11] Yuan Y., Cai X., Shen F., Ma F. (2021). HPV post-infection microenvironment and cervical cancer. Cancer Lett..

[12] Ajila V., Shetty H., Babu S., Shetty V., Hegde S. (2015). Human Papilloma Virus Associated Squamous Cell Carcinoma of the Head and Neck. J. Sex. Transm. Dis..

[13] Rantshabeng P.S., Moyo S., Moraka N.O., Ndlovu A., MacLeod I.J., Gaseitsiwe S., Kasvosve I. (2017). Prevalence of oncogenic human papillomavirus genotypes in patients diagnosed with anogenital malignancies in Botswana. BMC Infect. Dis..

[14] De Jong S.J., Imahorn E., Itin P., Uitto J., Orth G., Jouanguy E., Casanova J.-L., Burger B. (2018). Epidermodysplasia Verruciformis: Inborn Errors of Immunity to Human Beta-Papillomaviruses. Front. Microbiol..

[15] Deau M.C., Favre M., Jablonska S., Rueda L.A., Orth G. (1993). Genetic heterogeneity of oncogenic human papillomavirus type 5 (HPV5) and phylogeny of HPV5 variants associated with epidermodysplasia verruciformis. J. Clin. Microbiol..

[16] Orth G. (2008). Host Defenses against Human Papillomaviruses: Lessons from Epidermodysplasia Verruciformis. Curr. Top. Microbiol. Immunol..

[17] Béziat V. (2020). Human genetic dissection of papillomavirus-driven diseases: New insight into their pathogenesis. Hum. Genet..

[18] Béziat V., Casanova J.-L., Jouanguy E. (2021). Human genetic and immunological dissection of papillomavirus-driven diseases: New insights into their pathogenesis. Curr. Opin. Virol..

[19] Leiding J.W., Holland S.M. (2012). Warts and all: Human papillomavirus in primary immunodeficiencies. J. Allergy Clin. Immunol..

[20] Ramoz N., Rueda L.-A., Bouadjar B., Montoya L.-S., Orth G., Favre M. (2002). Mutations in two adjacent novel genes are associated with epidermodysplasia verruciformis. Nat. Genet..

[21] De Jong S.J., Créquer A., Matos I., Hum D., Gunasekharan V.K., Lorenzo L., Jabot-Hanin F., Imahorn E., Arias A.A., Vahidnezhad H. (2018). The human CIB1–EVER1–EVER2 complex governs keratinocyte-intrinsic immunity to β-papillomaviruses. J. Exp. Med..

[22] Atschekzei F., Jacobs R., Wetzke M., Sogkas G., Schröder C., Ahrenstorf G., Dhingra A., Ott H., Baumann U., Schmidt R.E. (2019). A Novel CARMIL2 Mutation Resulting in Combined Immunodeficiency Manifesting with Dermatitis, Fungal, and Viral Skin Infections as Well as Selective Antibody Deficiency. J. Clin. Immunol..

[23] Buschke A. (1896). Neisser’s Sterokopischer Atlas.

[24] Buschke A., Löwenstein L. (1925). Über carcinomähnliche condylomata accuminata des Penis. Klin. Wochenschr..

[25] Sabuncuoglu M.Z., Sabuncuoglu A., Celik G., Sozen I., Cetin R. (2014). Moist Secret Mass; Buschke-Lowenste in Tumor: A Report of Three Cases. Case Rep. Clin. Med..

[26] Chu Q.D., Vezeridis M.P., Libbey N.P., Wanebo H.J. (1994). Giant condyloma acuminatum (Buschke-Löwenstein tumor) of the anorectal and perianal regions. Analysis of 42 cases. Dis. Colon Rectum.

[27] Chan M.P. (2019). Verruciform and Condyloma-like Squamous Proliferations in the Anogenital Region. Arch. Pathol. Lab. Med..

[28] Sandhu R., Min Z., Bhanot N. (2014). A Gigantic Anogenital Lesion: Buschke-Lowenstein Tumor. Case Rep. Dermatol. Med..

[29] Davis K.G., Barton J.S., Orangio G., Bivin W., Krane S. (2021). Buschke-Lowenstein Tumors: A Review and Proposed Classification System. Sex. Transm. Dis..

[30] Zidar N., Langner C., Odar K., Hošnjak L., Kamarádová K., Daum O., Pollheimer M.J., Košorok P., Poljak M. (2017). Anal verrucous carcinoma is not related to infection with human papillomaviruses and should be distinguished from giant condyloma (Buschke-Löwenstein tumour). Histopathology.

[31] Haycox C.L., Kuypers J., Kriegerm J.N. (1999). Role of human papillomavirus typing in diagnosis and clinical decision making for a giant verrucous genital lesion. Urology.

[32] Pineda-Murillo J., Lugo-García J.A., Martínez-Carrillo G., Torres-Aguilar J., Viveros-Contreras C., Schettino-Peredo M.V. (2019). Buschke–Löwenstein tumor of the penis. Afr. J. Urol..

[33] Kadouri Y., Nouini Y. (2020). La tumeur de Buschke-Löwenstein [Buschke-Löwenstein’ tumor]. Pan Afr. Med. J..

[34] Bastola S., Halalau A., Kc O., Adhikari A. (2018). A Gigantic Anal Mass: Buschke–Löwenstein Tumor in a Patient with Controlled HIV Infection with Fatal Outcome. Case Rep. Infect. Dis..

[35] Diani M., Boneschi V., Ramoni S., Gadda F., Del Gobbo A., Cusini M. (2015). Rapidly Invasive Buschke-Löwenstein Tumor Associated with Human Papillomavirus Types 6 and 52. Sex. Transm. Dis..

[36] De Jong A.R., Weiss J.C., Brent R.L. (1982). Condyloma Acuminata in Children. Am. J. Dis..

[37] Nieves-Condoy J.F., Acuña-Pinzón C.L., Chavarría-Chavira J.L., Hinojosa-Ugarte D., Zúñiga-Vázquez L.A. (2021). Giant Condyloma Acuminata (Buschke-Lowenstein Tumor): Review of an Unusual Disease and Difficult to Manage. Infect. Dis. Obstet. Gynecol..

[38] Purzycka-Bohdan D., Szczerkowska-Dobosz A., Swiatecka-Czaj J., Peksa R., Urban M., Szczypior M., Nowicki R.J. (2019). Buschke-Löwenstein tumour associated with low-risk human papillomavirus genotypes successfully treated surgically. Postepy Dermatol. Allergol..

[39] El Khoury A., Jensen J.C., Pacioles T. (2019). Neoadjuvant chemotherapy and penile conservation in the management of Buschke–Lowenstein tumor, a case report. Urol. Case Rep..

[40] Combaud V., Verhaeghe C., El Hachem H., Legendre G., Descamps P., Martin L., Bouet P.-E. (2018). Giant condyloma acuminatum of the vulva: Successful management with imiquimod. JAAD Case Rep..

[41] Anissa Z., Houda H.-G., Wafa K., Olfa M., Rym B.-M., Achraf D., Samy F. (2015). Successful treatment with topical imiquimod of anal Buschke-Löwenstein tumor in a child. Dermatol. Ther..

[42] Dinleyici M., Saracoglu N., Eren M., Kiliç Ö., Ciftci E., Dinleyici E.C., Sag C., Kara A. (2015). Giant Condyloma Acuminate Due to Human Papillomavirus Type 16 in an Infant Successfully Treated with Topical Imiquimod Therapy. Dermatol. Rep..

[43] Hum M., Chow E., Schuurmans N., Dytoc M. (2018). Case of giant vulvar condyloma acuminata successfully treated with imiquimod 3.75% cream: A case report. SAGE Open Med. Case Rep..

[44] Geusau A., Heinz-Peer G., Volc-Platzer B., Stingl G., Kirnbauer R. (2000). Regression of Deeply Infiltrating Giant Condyloma (Buschke-Löwenstein Tumor) Following Long-term Intralesional Interferon Alfa Therapy. Arch. Dermatol..

[45] Petrini C.G., Melli P.P.D.S., Magnani P.S., Rocha L.P., Faria F.M., Duarte G., Quintana S.M. (2016). Giant Condyloma (Buschke-Loewenstein Tumor) in a 16-year-old Patient: Case Report. Rev. Bras. Ginecol. Obs..

[46] Grassegger A., Höpfl R., Hussl H., Wicke K., Fritsch P. (1994). Buschke—Loewenstein tumour infiltrating pelvic organs. Br. J. Dermatol..

[47] Antony F.C., Ardern-Jones M., Evans A.V., Rosenbaum T., Russell-Jones R. (2003). Giant condyloma of Buschke-Loewenstein in association with erythroderma. Clin. Exp. Dermatol..

[48] Bastard P., Michailidis E., Hoffmann H.-H., Chbihi M., Le Voyer T., Rosain J., Philippot Q., Seeleuthner Y., Gervais A., Materna M. (2021). Auto-antibodies to type I IFNs can underlie adverse reactions to yellow fever live attenuated vaccine. J. Exp. Med..

[49] Bustamante J. (2020). Mendelian susceptibility to mycobacterial disease: Recent discoveries. Hum. Genet..

[50] Duncan C.J., Mohamad S.M., Young D.F., Skelton A.J., Leahy T.R., Munday D.C., Butler K.M., Morfopoulou S., Brown J.R., Hubank M. (2015). Human IFNAR2 deficiency: Lessons for antiviral immunity. Sci. Transl. Med..

[51] Bowman I.A., Parra A., Arriaga Y. (2016). Metastatic Giant Condyloma Acuminata (Buschke-Löwenstein Tumor). J. Oncol. Pract..

[52] Lukács A., Máté Z., Farkas N., Mikó A., Tenk J., Hegyi P., Németh B., Czumbel L.M., Wuttapon S., Kiss I. (2020). The quadrivalent HPV vaccine is protective against genital warts: A meta-analysis. BMC Public Health.

[53] Kazlouskaya M., Fiadorchanka N. (2019). Regression of giant condyloma acuminata after one dose of 9-valent human papillomavirus (HPV) vaccine. Int. J. Dermatol..

[54] Thomas R., Smith-Matthews S., Ho J. (2021). Giant condyloma of Buschke-Lowenstein in a patient with pemphigus vegetans treated with intralesional and systemic human papillomavirus vaccine. JAAD Case Rep..

[55] McCutcheon T. (2009). Anal Condyloma Acuminatum. Gastroenterol. Nurs..

[56] Danielewski J.A., Garland S.M., McCloskey J., Hillman R.J., Tabrizi S.N. (2013). Human Papillomavirus Type 6 and 11 Genetic Variants Found in 71 Oral and Anogenital Epithelial Samples from Australia. PLoS ONE.

[57] Lin C., Franceschi S., Clifford G.M. (2018). Human papillomavirus types from infection to cancer in the anus, according to sex and HIV status: A systematic review and meta-analysis. Lancet Infect. Dis..

[58] Li N., Franceschi S., Howell-Jones R., Snijders P.J., Clifford G.M. (2011). Human papillomavirus type distribution in 30,848 invasive cervical cancers worldwide: Variation by geographical region, histological type and year of publication. Int. J. Cancer.

[59] Wells M., Robertson S., Lewis F., Dixon M.F. (1988). Squamous carcinoma arising in a giant peri-anal condyloma associated with human papillomavirus types 6 and 11. Histopathology.

[60] Martin J.M., Molina I., Monteagudo C., Martí N., Lopez V., Jorda E. (2008). Buschke-Lowenstein tumor. J. Dermatol. Case Rep..

[61] Handisurya A., Rieger A., Bago-Horvath Z., Schellenbacher C., Bankier A., Salat A., Stingl G., Kirnbauer R. (2009). Rapid progression of an anal Buschke-Lowenstein tumour into a metastasising squamous cell carcinoma in an HIV-infected patient. Sex. Transm. Infect..

[62] Egawa N., Doorbar J. (2017). The low-risk papillomaviruses. Virus Res..

[63] Hoppe-Seyler K., Bossler F., Braun J.A., Herrmann A.L., Hoppe-Seyler F. (2018). The HPV E6/E7 Oncogenes: Key Factors for Viral Carcinogenesis and Therapeutic Targets. Trends Microbiol..

[64] Vande Pol S.B., Klingelhutz A.J. (2013). Papillomavirus E6 oncoproteins. Virology.

[65] Roman A., Munger K. (2013). The papillomavirus E7 proteins. Virology.

[66] Mwapagha L.M., Tiffin N., Parker M.I. (2017). Delineation of the HPV11E6 and HPV18E6 Pathways in Initiating Cellular Transformation. Front. Oncol..

[67] Wu X., Xiao Y., Zhou S., Wang Y., Wang J. (2022). Transcriptomic Landscape of Gene Expression Profiles and Pathways in Juvenile-Onset Recurrent Respiratory Papillomatosis Tumor Tissues and Human Papillomavirus 6 and 11 E6- and E7-Overexpressing Head and Neck Squamous Cell Carcinoma Cell Lines. J. Virol..

[68] Murakami I., Egawa N., Griffin H., Yin W., Kranjec C., Nakahara T., Kiyono T., Doorbar J. (2019). Roles for E1-independent replication and E6-mediated p53 degradation during low-risk and high-risk human papillomavirus genome maintenance. PLoS Pathog..

[69] Gage J.R., Meyers C., Wettstein F.O. (1990). The E7 proteins of the nononcogenic human papillomavirus type 6b (HPV-6b) and of the oncogenic HPV-16 differ in retinoblastoma protein binding and other properties. J. Virol..

[70] Pilotti S., Donghi R., D’Amato L., Giarola M., Longoni A., Della Torre G., De Palo G., Pierotti M.A., Rilke F. (1993). HPV detection and p53 alteration in squamous cell verrucous malignancies of the lower genital tract. Diagn. Mol. Pathol..

[71] McBride A.A., Warburton A. (2017). The role of integration in oncogenic progression of HPV-associated cancers. PLoS Pathog..

[72] Péré H., Vernet R., Pernot S., Pavie J., Robillard N., Puech J., Lameiras S., Lucas M.-L., Nicolas A., Badoual C. (2021). Episomal HPV16 responsible for aggressive and deadly metastatic anal squamous cell carcinoma evidenced in peripheral blood. Sci. Rep..

[73] Cullen A.P., Reid R., Campion M., Lörincz A.T. (1991). Analysis of the physical state of different human papillomavirus DNAs in intraepithelial and invasive cervical neoplasm. J. Virol..

[74] Lehn H., Ernst T.-M., Sauer G. (1984). Transcription of Episomal Papillomavirus DNA in Human Condylomata Acuminata and Buschke-Löwenstein Tumours. J. Gen. Virol..

[75] Boshart M., zur Hausen H. (1986). Human papillomaviruses in Buschke-Löwenstein tumors: Physical state of the DNA and identification of a tandem duplication in the noncoding region of a human papillomavirus 6 subtype. J. Virol..

[76] Palefsky J.M., Giuliano A.R., Goldstone S., Moreira E.D., Aranda C., Jessen H., Hillman R., Ferris D., Coutlee F., Stoler M.H. (2011). HPV Vaccine against Anal HPV Infection and Anal Intraepithelial Neoplasia. N. Engl. J. Med..

[77] Insinga R.P., Liaw K.-L., Johnson L.G., Madeleine M.M. (2008). A Systematic Review of the Prevalence and Attribution of Human Papillomavirus Types among Cervical, Vaginal, and Vulvar Precancers and Cancers in the United States. Cancer Epidemiol. Biomark. Prev..

[78] Manias D.A., Ostrow R.S., McGlennen R.C., Estensen R.D., Faras A.J. (1989). Characterization of integrated human papillomavirus type 11 DNA in primary and metastatic tumors from a renal transplant recipient. Cancer Res..

[79] Reidy P.M., Dedo H.H., Rabah R., Field J.B., Mathog R.H., Gregoire L., Lancaster W.D. (2004). Integration of Human Papillomavirus Type 11 in Recurrent Respiratory Papilloma-Associated Cancer. Laryngoscope.

[80] Bercovich J.A., Centeno C.R., Aguilar O.G., Grinsteinm S., Kahn T. (1991). Presence and integration of human papillomavirus type 6 in a tonsillar carcinoma. J. Gen. Virol..

[81] Kahn T., Turazza E., Ojeda R., Bercovich A., Stremlau A., Lichter P., Poustka A., Grinstein S., zur Hausen H. (1994). Integration of human papillomavirus type 6a DNA in a tonsillar carcinoma: Chromosomal localization and nucleotide sequence of the genomic target region. Cancer Res..

[82] Rübben A., Beaudenon S., Favre M., Schmitz W., Spelten B., Grussendorf-Conen E.-I. (1992). Rearrangements of the upstream regulatory region of human papillomavirus type 6 can be found in both Buschke-Löwenstein tumours and in condylomata acuminata. J. Gen. Virol..

[83] Albert R., Spelten B., Albrecht J., Grußendorf-Conen E.-I. (1994). Demonstration of URR-duplication variants of human papillomavirus type 6 in paraffin-embedded tissue sections of one condyloma acuminatum and one buschke-lowenstein tumour. J. Pathol..

[84] Kitasato H., Delius H., zur Hausen H., Sorger K., Rösl F., de Villiers E.-M. (1994). Sequence Rearrangements in the Upstream Regulatory Region of Human Papillomavirus Type 6: Are these Involved in Malignant Transition?. J. Gen. Virol..

[85] Rosen M., Auborn K. (1991). Duplication of the upstream regulatory sequences increases the transformation potential of human papillomavirus type 11. Virology.

[86] Di Luca D., Caselli E., Monini P., Rotola A., Savioli A., Cassai E. (1989). Episomal HPV 16 DNA isolated from a cervical carcinoma presents a partial duplication of the early region. Virus Res..

[87] Byrne J.C., Tsao M.S., Fraser R.S., Howley P.M. (1987). Human Papillomavirus-11 DNA in a Patient with Chronic Laryngotracheobronchial Papillomatosis and Metastatic Squamous-Cell Carcinoma of the Lung. N. Engl. J. Med..

[88] DiLorenzo T.P., Tamsen A., Abramson A.L., Steinberg B.M. (1992). Human papillomavirus type 6a DNA in the lung carcinoma of a patient with recurrent laryngeal papillomatosis is characterized by a partial duplication. J. Gen. Virol..

[89] Kasher M.S., Roman A. (1988). Characterization of human papillomavirus type 6b DNA isolated from an invasive squamous carcinoma of the vulva. Virology.

[90] Rando R.F., Groff D.E., Chirikjian J.G., Lancaster W.D. (1986). Isolation and characterization of a novel human papillomavirus type 6 DNA from an invasive vulvar carcinoma. J. Virol..

[91] Rando R.F., Lancaster W.D., Han P., Lopez C. (1986). The noncoding region of HPV-6vc contains two distinct transcriptional enhancing elements. Virology.

[92] Farr A., Wang H., Kasher M.S., Roman A. (1991). Relative enhancer activity and transforming potential of authentic human papillomavirus type 6 genomes from benign and malignant lesions. J. Gen. Virol..

[93] Sporkert M., Rübben A. (2017). Buschke-Löwenstein-Tumor [Buschke-Lowenstein tumors]. Hautarzt.

[94] Wieland U., Kreuter A., Pfister H. (2014). Human Papillomavirus and Immunosuppression. Curr. Probl. Dermatol..

[95] Chin-Hong P.V., Reid G.E., The AST Infectious Diseases Community of Practice (2019). Human papillomavirus infection in solid organ transplant recipients: Guidelines from the American Society of Transplantation Infectious Diseases Community of Practice. Clin. Transplant..

[96] Gormley R.H., Kovarik C.L. (2012). Human papillomavirus–related genital disease in the immunocompromised host: Part I. J. Am. Acad. Dermatol..

[97] Tschachler E., Bergstresser P.R., Stingl G. (1996). HIV-related skin diseases. Lancet.

[98] Coleman N., Birley H.D., Renton A.M., Hanna N.F., Ryait B.K., Byrne M., Taylor-Robinson D., Stanley M.A. (1994). Immunological Events in Regressing Genital Warts. Am. J. Clin. Pathol..

[99] Fierlbeck G., Schiebel U., Müller C. (1989). Immunohistology of Genital Warts in Different Stages of Regression after Therapy with Interferon Gamma. Dermatology.

[100] Akinboro A.O., Onayemi O., Mejiuni A.D. (2014). Frequency, pattern, and extent of skin diseases in relation to CD4+ cell count among adults with human immunodeficiency virus infection or acquired immunodeficiency syndrome in Osogbo, southwestern Nigeria. Int. J. Dermatol..

[101] Reusser N.M., Downing C., Guidry J., Tyring S.K. (2015). HPV Carcinomas in Immunocompromised Patients. J. Clin. Med..

[102] Dhumale S.B. (2017). Ano-Genital Warts and HIV Status–A Clinical Study. J. Clin. Diagn. Res..

[103] De Araújo P.S.R., Padilha C.E.G., Soares M.F. (2017). Buschke-Lowenstein tumor in a woman living with HIV/AIDS. Rev. Soc. Bras. Med. Trop..

[104] Atkinson A.L., Pursell N., Sisay A. (2014). The Giant Condyloma (Buschke-Löwenstein Tumor) in the Immunocompromised Patient. Case Rep. Obstet. Gynecol..

[105] Ledouble V., Sclafani F., Hendlisz A., Galdon M.G., Liberale G. (2021). Buschke-Löwenstein tumor in a human immunodeficiency virus-positive patient: A case report and short literature review. Acta Gastroenterol. Belg..

[106] Rachman A., Hasan N. (2016). Giant Condyloma Acuminata in Indonesian Females with SLE under Immunosuppressant and Steroid Therapy. Case Rep. Immunol..

[107] Ganguly N., Waller S., Stasik C.J., Skiknem B.S., Ganguly S. (2008). Giant anal condylomatosis after allogeneic bone marrow transplantation: A rare complication of human papilloma virus infection. Transpl. Infect. Dis..

[108] Das B.B., Anton K., Knox L., Jarin J., Sue P.K. (2018). Successful treatment of giant condyloma in a pediatric heart transplant recipient with topical cidofovir. Transpl. Infect. Dis..

[109] Wester N.E., Hutten E.M., Krikke C., Pol R.A. (2013). Intra-Abdominal Localisation of a Buschke-Lowenstein Tumour: Case Presentation and Review of the Literature. Case Rep. Transplant..

[110] Indinnimeo M., Impagnatiello A., D’Ettorre G., Bernardi G., Moschella C.M., Gozzo P., Ciardi A., Bangrazi C., De Felice F., Musio D. (2013). Buschke-Löwenstein tumor with squamous cell carcinoma treated with chemo-radiation therapy and local surgical excision: Report of three cases. World J. Surg. Oncol..

[111] Grodner C., Henn A., Lelièvre J.-D., Gallien S. (2016). Successful improvement of Buschke-Löwenstein tumour in an HIV-infected patient with antiretroviral therapy alone. BMJ Case Rep..

[112] Wang Y., Ma C.S., Ling Y., Bousfiha A., Camcioglu Y., Jacquot S., Payne K., Crestani E., Roncagalli R., Belkadi A. (2016). Dual T cell– and B cell–intrinsic deficiency in humans with biallelic RLTPR mutations. J. Exp. Med..

[113] Sorte H.S., Osnes L.T., Fevang B., Aukrust P., Erichsen H.C., Backe P.H., Abrahamsen T.G., Kittang O.B., Øverland T., Jhangiani S. (2016). A potential founder variant inCARMIL2/RLTPRin three Norwegian families with warts, molluscum contagiosum, and T-cell dysfunction. Mol. Genet. Genom. Med..

[114] Meuris F., Carthagena L., Jaracz-Ros A., Gaudin F., Cutolo P., Deback C., Xue Y., Thierry F., Doorbar J., Bachelerie F. (2016). The CXCL12/CXCR4 Signaling Pathway: A New Susceptibility Factor in Human Papillomavirus Pathogenesis. PLoS Pathog..

[115] Handisurya A., Schellenbacher C., Reininger B., Koszik F., Vyhnanek P., Heitger A., Kirnbauer R., Förster-Waldl E. (2010). A quadrivalent HPV vaccine induces humoral and cellular immune responses in WHIM immunodeficiency syndrome. Vaccine.

[116] McDermott D.H., Murphy P.M. (2019). WHIM syndrome: Immunopathogenesis, treatment and cure strategies. Immunol. Rev..

[117] Woodbine L., Grigoriadou S., Goodarzi A.A., Riballo E., Tape C., Oliver A.W., Van Zelm M.C., Buckland M.S., Davies E.G., Pearl L.H. (2010). An Artemis polymorphic variant reduces Artemis activity and confers cellular radiosensitivity. DNA Repair.

[118] Volk T., Pannicke U., Reisli I., Bulashevska A., Ritter J., Björkman A., Schäffer A.A., Fliegauf M., Sayar E.H., Salzer U. (2015). *DCLRE1C*(ARTEMIS) mutations causing phenotypes ranging from atypical severe combined immunodeficiency to mere antibody deficiency. Hum. Mol. Genet..

[119] Chu E.Y., Freeman A.F., Jing H., Cowen E.W., Davis J., Su H.C., Holland S.M., Turner M.L. (2012). Cutaneous Manifestations of DOCK8 Deficiency Syndrome. Arch. Dermatol..

[120] Al-Zahrani D., Raddadi A., Massaad M., Keles S., Jabara H.H., Chatila T.A., Geha R. (2014). Successful interferon-alpha 2b therapy for unremitting warts in a patient with DOCK8 deficiency. Clin. Immunol..

[121] Venegas-Montoya E., Staines-Boone A.T., Sánchez-Sánchez L.M., García-Campos J.A., Córdova-Gurrola R.A., Salazar-Galvez Y., Múzquiz-Zermeño D., González-Serrano M.E., Reyes S.O.L. (2021). Case Report: DOCK8 Deficiency without Hyper-IgE in a Child with a Large Deletion. Front. Pediatr..

[122] Hsu A.P., McReynolds L.J., Holland S.M. (2015). GATA2 deficiency. Curr. Opin. Allergy Clin. Immunol..

[123] Spinner M.A., Sanchez L.A., Hsu A.P., Shaw P.A., Zerbe C.S., Calvo K.R., Arthur D.C., Gu W., Gould C.M., Brewer C.C. (2014). GATA2 deficiency: A protean disorder of hematopoiesis, lymphatics, and immunity. Blood.

[124] Kuriyama Y., Hattori M., Mitsui T., Nakano H., Oikawa D., Tokunaga F., Ishikawa O., Shimizu A. (2018). Generalized verrucosis caused by various human papillomaviruses in a patient with GATA2 deficiency. J. Dermatol..

[125] Cole K., Avila D., Parta M., Schuver B., Holland S., Shah N., Hickstein D. (2019). GATA2 Deficiency: Early Identification for Improved Clinical Outcomes. Clin. J. Oncol. Nurs..

[126] Roussel L., Landekic M., Golizeh M., Gavino C., Zhong M.-C., Chen J., Faubert D., Blanchet-Cohen A., Dansereau L., Parent M.-A. (2018). Loss of human ICOSL results in combined immunodeficiency. J. Exp. Med..

[127] van de Vijver E., Maddalena A., Sanal Ö., Holland S.M., Uzel G., Madkaikar M., de Boer M., van Leeuwen K., Köker M.Y., Parvaneh N. (2012). Hematologically important mutations: Leukocyte adhesion deficiency (first update). Blood Cells Mol. Dis..

[128] Ravell J.C., Matsuda-Lennikov M., Chauvin S.D., Zou J., Biancalana M., Deeb S.J., Price S., Su H.C., Notarangelo G., Jiang P. (2020). Defective glycosylation and multisystem abnormalities characterize the primary immunodeficiency XMEN disease. J. Clin. Investig..

[129] Li A.L., Walsh S., McKay D.R. (2011). Surgical management of a giant condyloma of Buschke-Löwenstein in a patient with Netherton syndrome using the pedicled anterolateral thigh flap—A case report. J. Plast. Reconstr. Aesthetic Surg..

[130] Ashton R., Moledina J., Sivakumar B., Mellerio J.E., Martinez A.E. (2017). Considerations in surgical management of a Buschke-Lowenstein tumor in Netherton syndrome: A case report. Pediatr. Dermatol..

[131] Mehta H., Paz J.C., Sadikot R.T. (2008). Wiskott–Aldrich syndrome with bronchiectasis. Respir. Med. CME.

[132] Kawai T., Malech H.L. (2009). WHIM syndrome: Congenital immune deficiency disease. Curr. Opin. Hematol..

[133] Donadieu J., Lamant M., Fieschi C., De Fontbrune F.S., Caye A., Ouachee M., Beaupain B., Bustamante J., Poirel H.A., Isidor B. (2018). Natural history of GATA2 deficiency in a survey of 79 French and Belgian patients. Haematologica.

[134] Zhang Q., Dove C.G., Hor J.L., Murdock H.M., Strauss-Albee D.M., Garcia J.A., Mandl J.N., Grodick R.A., Jing H., Chandler-Brown D.B. (2014). DOCK8 regulates lymphocyte shape integrity for skin antiviral immunity. J. Exp. Med..

[135] Harada Y., Tanaka Y., Terasawa M., Pieczyk M., Habiro K., Katakai T., Hanawa-Suetsugu K., Kukimoto-Niino M., Nishizaki T., Shirouzu M. (2012). DOCK8 is a Cdc42 activator critical for interstitial dendritic cell migration during immune responses. Blood.

[136] Dorjbal B., Stinson J.R., Ma C.A., Weinreich M.A., Miraghazadeh B., Hartberger J.M., Frey-Jakobs S., Weidinger S., Moebus L., Franke A. (2019). Hypomorphic caspase activation and recruitment domain 11 (CARD11) mutations associated with diverse immunologic phenotypes with or without atopic disease. J. Allergy Clin. Immunol..

[137] Chinn I.K., Sanders R.P., Stray-Pedersen A., Coban-Akdemir Z.H., Kim V.H.-D., Dadi H., Roifman C.M., Quigg T., Lupski J.R., Orange J.S. (2017). Novel Combined Immune Deficiency and Radiation Sensitivity Blended Phenotype in an Adult with Biallelic Variations in ZAP70 and RNF168. Front. Immunol..

[138] Schepp J., Chou J., Skrabl-Baumgartner A., Arkwright P.D., Engelhardt K.R., Hambleton S., Morio T., Röther E., Warnatz K., Geha R.S. (2017). 14 Years after Discovery: Clinical Follow-up on 15 Patients with Inducible Co-Stimulator Deficiency. Front. Immunol..

[139] Insinga R.P., Dasbach E.J., Myers E.R. (2003). The Health and Economic Burden of Genital Warts in a Set of Private Health Plans in the United States. Clin. Infect. Dis..

[140] Lipke M.M. (2006). An Armamentarium of Wart Treatments. Clin. Med. Res..

